# Global landscape and professionalization of infection preventionist education: comparative insights and future directions

**DOI:** 10.3389/fpubh.2025.1736787

**Published:** 2026-01-09

**Authors:** Yu-long Cao, Jiao Shan, Xiao-yuan Bao, Meng Jin, Wei Huai, Yi-cheng Jin, Yi-xi Jin, Ze-xin Zhang, Ji-qiu Kuang

**Affiliations:** 1 Department of Hospital-Acquired Infection Control, Peking University People's Hospital, Beijing, China; 2 Department of Hospital-Acquired Infection Control, Beijing Jishuitan Hospital, Capital Medical University, Beijing, China; 3 Medical Informatics Center, Institute of Advanced Clinical Medicine, Peking University, Beijing, China; 4 Department of Emergency, Peking University Third Hospital, Beijing, China; 5 School of General Studies, Columbia University, New York, NY, United States; 6 Khoury College of Computer Science, Northeastern University, Seattle, WA, United States; 7 Graduate School of Medicine, Kyoto University, Kyoto, Japan

**Keywords:** infection control education, training model, infection preventionist, certification, competency framework

## Abstract

Healthcare-associated infections (HAIs) continue to pose a significant threat to global public health, underscoring the critical role of infection preventionists (IPs). However, considerable heterogeneity exists in IP training and credentialing worldwide. This mini-review synthesizes existing models from diverse healthcare systems into a unified “IP Education Maturity Spectrum.” This conceptual framework categorizes global training systems into Nascent, Developing, and Established stages, defined by their maturity across three dimensions: Institutional Integration, Competency Orientation, and Career Sustainability. Furthermore, we address the “Professional Identity Paradox”—the structural tension between the field’s multidisciplinary roots and the need for a specialized professional identity. Finally, we position academic integration, digital education, and global harmonization not merely as future trends, but as strategic interventions designed to propel systems up the maturity spectrum toward full professionalization.

## 1 Introduction

Healthcare-associated infections (HAIs) remain one of the most enduring and consequential challenges in modern healthcare, contributing substantially to morbidity, mortality, and healthcare costs globally (1). Effective infection prevention and control (IPC) programs are widely acknowledged as fundamental pillars of patient safety and clinical quality (2). Infection preventionists (IPs) are central to the success of IPC programs, serving as specialized professionals who design, implement, and evaluate strategies to mitigate infection risks in healthcare environments (3). As healthcare systems grow increasingly complex and emerging infectious diseases continue to pose new threats, the need for a competent, well-trained, and standardized IPs workforce has become more urgent than ever before (4).

Despite the global recognition of IPC as a core healthcare function, substantial heterogeneity persists in the ways IPs are trained, certified, and integrated into healthcare systems. In several high-income countries—such as the United States, the United Kingdom, and Japan—well-defined competency frameworks and formal certification mechanisms have been established, underpinned by professional organizations and academic institutions (5). In contrast, in many parts of Asia and in low- and middle-income countries, IPs are often trained through in-service or *ad hoc* programs, typically lacking standardized curricula or formal accreditation frameworks (6). Such variations in educational pathways and professional recognition can result in inconsistent competencies and constrained professional mobility among IPs across regions.

The COVID-19 pandemic further exposed and amplified these disparities. Countries with standardized and tiered IPC training systems exhibited greater resilience, coordination, and adaptability in their outbreak responses, whereas regions lacking such infrastructure struggled to mobilize an adequately trained infection prevention workforce (7). Understanding how different countries structure their IP education and certification systems offers critical insights for developing a more resilient, standardized, and globally aligned IPC workforce.

## 2 The integrated framework: the IP education maturity Spectrum

To systematically map the global landscape, the “IP Education Maturity Spectrum” utilizes three non-linear operational dimensions to define a country’s developmental stage, providing a comprehensive assessment tool for comparative analysis.

The first dimension is Institutional Integration, which measures the degree to which IP education and training are formally embedded within the national academic and regulatory infrastructure (8). At the low end, training is ad-hoc and episodic, relying heavily on temporary funding or short-term workshops (9). At the high end, IP education is offered through specialized, accredited Master’s or Doctoral programs, fully integrated into university systems and mandated by national health policy. High integration suggests stability and permanence in the educational pipeline (10).

The second dimension, Competency Orientation, examines the foundation of the IP curriculum. Lower maturity levels are characterized by task-based or check-list training, often lacking the theoretical depth necessary for complex decision-making and quality improvement science (11). Higher maturity levels mandate framework-driven knowledge acquisition, where education is based on defined, rigorous professional competencies (e.g., the APIC or IFIC models) that require advanced knowledge in epidemiology, microbiology, and process improvement methodology (12, 13).

The final dimension, Career Sustainability, is perhaps the most critical indicator of professional maturity. It evaluates the existence of a clear, attractive professional pathway, including formalized certification, compensation linked to credentials, and defined leadership roles. Low sustainability leads to high attrition rates and undermines expertise accumulation (14). Conversely, high sustainability ensures the IP role is viewed as a primary, specialized career—one with clear potential for advancement and influence within the healthcare hierarchy (15). Within this framework, a system’s combined performance across these three dimensions determines its classification into the Nascent, Developing, or Established Stage.

## 3 The global landscape analysis: the stages of professionalization

### 3.1 Stage 1: the nascent stage

The Nascent Stage is primarily defined by critically low scores across the three dimensions, particularly in Institutional Integration and Career Sustainability. Geographically, this stage frequently characterizes many low- and middle-income countries (LMICs), notably in Sub-Saharan Africa and parts of South Asia. In these regions, the primary driver for IP training is often reactive, stemming from crises such as the Ebola or COVID-19 pandemics, or initiated by external non-governmental or multilateral organizations (16). Training is typically delivered through workshops or short-term programs, such as those associated with Field Epidemiology Training Programs (FELTPs), which, while valuable for acute response, are not institutionally permanent (17).

A specific example of this challenge is the near-total reliance on donor-driven funding, which often makes training episodic and unsustainable. Once external funding wanes, the infrastructure—including trained mentors and specialized faculty—often collapses, leading to a profound gap in the educational pipeline. Furthermore, the IP role in this stage is often not codified as a distinct profession. Instead, the task is typically “borrowed” by a nurse or clinician, for which they receive little to no additional compensation or career advancement (18). This structural deficiency in Career Sustainability leads directly to high turnover, preventing the essential accumulation of experienced professionals and trapping the system in a perpetual cycle of basic, reactive training. Addressing this stage requires immediate intervention to stabilize the educational base and foster local capacity that is not dependent on external actors (19).

### 3.2 Stage 2: the developing stage (transitional professionalization)

The Developing Stage represents systems that have achieved partial maturity, demonstrating moderate Institutional Integration but struggling profoundly with the Professional Identity Paradox and inconsistent Competency Orientation. This category includes major systems across Latin America, the Middle East, and emerging economies like China. In these contexts, formal regulatory requirements exist, and tertiary institutions may offer postgraduate diplomas or certificates (20).

However, the field is deeply affected by the structural tension inherent in its multidisciplinary origins. Because the IP role is often filled by individuals predominantly trained in Nursing or Public Health—as is common in Brazil or Middle Eastern countries where nursing forms the IP backbone—a fundamental ambiguity exists (21). Is the goal to transition to a singular, specialized IP identity, or to broadly enhance IP competency across multiple foundational professions? This Professional Identity Paradox creates tension in practice. For example, in China, while IPs may hold specialist responsibilities, their official job code remains tied to clinical nursing or laboratory staff, meaning they lack the regulatory autonomy and pay grade commensurate with their specialized function (22). This structural limitation compromises Competency Orientation because the absence of a standardized, regulated IP status means that competency assessments, while present, lack the legal and professional leverage to enforce continuous, advanced professional development. Ultimately, this stage is characterized by a “glass ceiling” on professional influence, where the IP role is valued, but not fully recognized or structurally rewarded.

### 3.3 Stage 3: the established stage (full professionalization)

The Established Stage represents systems that have achieved high maturity across all three dimensions, notably including the United States, Germany, the United Kingdom, and Japan. These systems have successfully resolved the Professional Identity Paradox by institutionalizing the IP role. Institutional Integration is robust (23). IP education is provided through accredited academic programs at the graduate level, often mandated by professional boards.

The defining characteristic of this stage is high Competency Orientation. Education is guided by dynamic, regularly updated competency models (e.g., the APIC Competency Model, IFIC Professional Framework), and achievement is validated through rigorous, independent certification (e.g., the Certification in Infection Control, CIC®) (24). Crucially, Career Sustainability is maximized: certification is directly linked to compensation scales, eligibility for senior leadership positions, and professional mobility. For example, the Japanese Certified Nurse in Infection Control (CNIC) program provides a clear pathway for nurses to gain specialized authority and prestige, securing the IP profession’s position as a distinct and indispensable specialty (25). The longevity and influence of IP leadership in these countries demonstrate the effectiveness of this fully integrated model.

## 4 Strategic interventions for professionalization

The transition between stages on the Maturity Spectrum is not passive. it requires deliberate, targeted strategic interventions that address the specific structural deficits identified in the analysis.

### 4.1 Academic integration: solving the professional identity paradox

The most critical structural challenge is the Professional Identity Paradox prevalent in the Developing Stage. Attributing this issue solely to “poor training” is insufficient; the problem is structural. The intervention requires moving beyond certificate programs to formalizing IP as a distinct academic discipline. Academic Integration entails establishing interdisciplinary graduate programs that are explicitly open to professionals from Public Health, Nursing, and Laboratory Science alike (26). This strategy serves two functions: First, it standardizes the theoretical foundation regardless of the entering discipline, and second, it creates a regulatory foundation for the infection preventionist job code (27). By establishing IP as a recognized academic specialization, the field secures a permanent and respected position within the health system, resolving the tension and creating genuine Career Sustainability.

### 4.2 Digital education ecosystems: bridging the institutional integration gap

To address the profound lack of Institutional Integration and resource constraints characteristic of the Nascent Stage, Digital Education Ecosystems offer a vital, scalable solution. This intervention involves utilizing high-quality e-learning modules, virtual simulation laboratories, and low-bandwidth learning platforms to decouple rigorous training from the need for centralized, physical university infrastructure (28). Evidence from pilot programs in South Asia has shown that competency-based online modules can effectively deliver advanced IPC knowledge to large cohorts of nurses and allied health workers in low-resource settings (29). This strategy directly impacts Institutional Integration by providing a mechanism to rapidly standardize workforce training and establish measurable competency benchmarks without awaiting the slow maturation of traditional academic structures. This allows Nascent systems to leapfrog certain infrastructural obstacles.

### 4.3 Global harmonization: solving the competency and mobility barriers

While Established systems have high internal standardization, Global Harmonization is required to ensure interoperability across the Maturity Spectrum. This intervention targets the Competency Orientation and Career Sustainability dimensions globally. Harmonization does not mandate identical curricula; instead, it requires the mutual recognition of core competencies, ensuring that an IP certified in a Developing system meets a universally accepted baseline. Mapping studies across various European nations have already demonstrated the complexity and necessity of this process. Adopting global standards facilitates professional mobility, allowing experienced IPs to contribute effectively across borders during global health crises (11). It also provides a clear, aspirational benchmark for Developing and Nascent systems, focusing their educational efforts on achieving these internationally recognized competencies, thereby strengthening overall global health security readiness.

## 5 Conclusion

The professionalization of the IP workforce is not a passive process but a strategic progression along the IP Education Maturity Spectrum. The path to full professionalization requires stakeholders to first acknowledge and systematically map their system’s deficits across Institutional Integration, Competency Orientation, and Career Sustainability. The key structural bottleneck—the Professional Identity Paradox—must be resolved through Academic Integration. Furthermore, leveraging the scalability of Digital Education Ecosystems is crucial for bridging infrastructural gaps in Nascent systems, while Global Harmonization ensures competency alignment across the entire spectrum. Ultimately, advancing national IP education systems is not merely an academic endeavor, but a fundamental prerequisite for mitigating HAI risk and securing global.

## Data Availability

All data used in this review are derived from publicly available sources, including PubMed-indexed articles and official publications from professional organizations.

## References

[1] World Health Organization. Global strategy on infection prevention and control. Geneva, Switzerland: World Health Organization (2024).

[2] Tartari E Tomczyk S Twyman A Rehse APC Gomaa M Talaat M . Evaluating national infection prevention and control minimum requirements: evidence from global cross-sectional surveys, 2017-22. Lancet Glob Health. (2024) 12:e1620–8. doi: 10.1016/S2214-109X(24)00277-8, 39304235 PMC11420467

[3] Abbas S. The challenges of implementing infection prevention and antimicrobial stewardship programs in resource-constrained settings. Antimicrobial Stewardship Healthcare Epidemiology. (2024) 4:e45. doi: 10.1017/ash.2024.35, 38628374 PMC11019578

[4] Jeong SY Kim OS. Development and application of an educational-training programme for infection control practitioners in long-term care hospitals. Healthcare (Basel). (2023) 11:542. doi: 10.3390/healthcare11040542, 36833076 PMC9957071

[5] da Silva Felix AM Pereira EG Padoveze MC. Competency assessment tools for infection preventionists: a scoping review. J Infect Prev. (2023) 24:259–67. doi: 10.1177/17571774231203388, 37975067 PMC10638954

[6] Tomczyk S Storr J Kilpatrick C Allegranzi B. Infection prevention and control (IPC) implementation in low-resource settings: a qualitative analysis. Antimicrob Resist Infect Control. (2021) 10:113. doi: 10.1186/s13756-021-00962-3, 34332622 PMC8325287

[7] World Health Organization. Core competencies for infection prevention and control professionals. 1st ed. Geneva: World Health Organization (2020).

[8] Allegranzi B Kilpatrick C Storr J Kelley E Park BJ Donaldson L . Global infection prevention and control priorities 2018-22: a call for action. Lancet Glob Health. (2017) 5:e1178–80. doi: 10.1016/S2214-109X(17)30427-8, 29132606 PMC7129117

[9] Cao Y Shan J Gong Z Kuang J Gao Y. Status and challenges of public health emergency management in China related to COVID-19. Front Public Health. (2020) 8:250. doi: 10.3389/fpubh.2020.00250, 32574311 PMC7273973

[10] Holmes K Boston KM McCarty J Steinfeld S Kennedy V. Enhancing infection preventionist certification success through a structured training program. Am J Infect Control. (2024) 52:1235–40. doi: 10.1016/j.ajic.2024.07.015, 39089492

[11] Sowar SF Acunin R Abo Arisheh T Cabanalan HC Alkhawaja S. Evaluation of infection preventionists’ competencies using the association for professionals in infection control and epidemiology competency model in tertiary care government hospitals, Bahrain. Cureus. (2024) 16:e65764. doi: 10.7759/cureus.65764, 39211652 PMC11361617

[12] Hrzic R Cade MV Wong BLH McCreesh N Simon J Czabanowska K. A competency framework on simulation modelling-supported decision-making for master of public health graduates. J Public Health. (2024) 46:127–35. doi: 10.1093/pubmed/fdad248, 38061776 PMC10901273

[13] Talbot TR Baliga C Crapanzano-Sigafoos R Bubb TN Fakih M Fraser TG . SHEA/APIC/IDSA/PIDS multisociety position paper: raising the bar: necessary resources and structure for effective healthcare facility infection prevention and control programs. Infect Control Hosp Epidemiol. (2025) 46:665–83. doi: 10.1017/ice.2025.73, 40289573 PMC12277079

[14] Ni L Yin X Liu L Sun K Zhang W. Research on the effectiveness of the training of nosocomial infection control specialist nurses under the background of the new crown epidemic based on competence-based theory. Altern Ther Health Med. (2024) AT10298. 38758138

[15] Tsioutis C Birgand G Bathoorn E Deptula A Ten Horn L Castro-Sánchez E . Education and training programmes for infection prevention and control professionals: mapping the current opportunities and local needs in european countries. Antimicrob Resist Infect Control. (2020) 9:183. doi: 10.1186/s13756-020-00835-1, 33168085 PMC7652580

[16] Kim Y Gwack J Kwon Y Cheong MJ Lee J-H. Needs assessment for public health competency in infection prevention and control: importance and performance analysis (IPA) of infectious disease response practitioners. J Korean Med Sci. (2025) 40:e23. doi: 10.3346/jkms.2025.40.e23, 39763309 PMC11707661

[17] Alshamrani MM Tannous E Othman F Al Zunitan M Abalkhail M El-Saed A. Competency level and determinants among infection prevention and control staff in the middle east and North Africa region. BMC Public Health. (2024) 24:2224. doi: 10.1186/s12889-024-19717-x, 39148097 PMC11328368

[18] Wang H. Public health emergency decision-making and management system sound research using rough set attribute reduction and blockchain. Sci Rep. (2022) 12:3600. doi: 10.1038/s41598-022-07493-w, 35246582 PMC8897403

[19] Kranenburg LW de Veer MR Oude Hengel KM Kouwenhoven-Pasmooij TA de Pagter AP Hoogendijk WJ . Need for support among healthcare professionals during the COVID-19 pandemic: a qualitative study at an academic hospital in the Netherlands. BMJ Open. (2022) 12:e059124. doi: 10.1136/bmjopen-2021-059124, 35210349 PMC8882635

[20] Hill B Lamichhane G Wamburu A. Infection prevention and control: critical strategies for nursing practice. Br J Nurs. (2024) 33:804–11. doi: 10.12968/bjon.2024.0286, 39302906

[21] Fabre V Secaira C Herzig C Bancroft E Bernachea MP Galarza LA . Contextual barriers to infection prevention and control program implementation in hospitals in latin america: a mixed methods evaluation. Antimicrob Resist Infect Control. (2024) 13:132. doi: 10.1186/s13756-024-01484-4, 39491033 PMC11533356

[22] Tu R Zhao T Yu X Xiao Z Zhou J Tian J . Assessment of infection prevention and control management in obstetrics departments in secondary and tertiary hospitals: a cross-sectional study from Guiyang city, China, 2023. BMC Infect Dis. (2025) 25:383. doi: 10.1186/s12879-025-10598-y, 40108524 PMC11924702

[23] Tingelhoff P-D Hufert F Kiessling C Otto B. Infection prevention in medical education - results of a descriptive cross-sectional study in Germany. GMS J Med Educ. (2024) 41:Doc4. doi: 10.3205/zma001659, 38504860 PMC10946213

[24] Kubde D Badge AK Ugemuge S Shahu S. Importance of hospital infection control. Cureus. (2023) 15:e50931. doi: 10.7759/cureus.50931, 38259418 PMC10801286

[25] Kawakami K Misao H. Developing a competency model for nurses certified in infection control in Japan. Infect Control Hosp Epidemiol. (2020) 41:s514–5. doi: 10.1017/ice.2020.1196

[26] Gorjian Z Asadizaker M Zarea K Irajpour A Ahmadi F Rokhafroz D. A comparative study of infection prevention and control curricula in nursing master’s degree programs worldwide, with practical suggestions for Iran. J Educ Health Promot. (2025) 14:71. doi: 10.4103/jehp.jehp_1723_23, 40144169 PMC11940058

[27] Al-Tawfiq JA. Striving for zero traditional and non-traditional healthcare-associated infections (HAI): a target, vision, or philosophy. Antimicrobial Stewardship Healthcare Epidemiology. (2025) 5:e146. doi: 10.1017/ash.2025.10031, 40612460 PMC12224139

[28] Anglemyer A Moore TH Parker L Chambers T Grady A Chiu K . Digital contact tracing technologies in epidemics: a rapid review. Cochrane Database Syst Rev. (2020) 2020:CD013699. doi: 10.1002/14651858.CD013699, 33502000 PMC8241885

[29] Sayami JT Amatya R Karki K Bajracharya D Shrestha B Srinivasan S . A nursing and midwifery training program in Kathmandu on antimicrobial resistance and stewardship and infection prevention and control: a qualitative and quantitative outcomes and process evaluation. Front Public Health. (2025) 13:1497335. doi: 10.3389/fpubh.2025.1497335, 39916710 PMC11799561

